# Elevated CSF inflammatory markers in patients with idiopathic normal pressure hydrocephalus do not promote NKCC1 hyperactivity in rat choroid plexus

**DOI:** 10.1186/s12987-021-00289-6

**Published:** 2021-12-04

**Authors:** Sara Diana Lolansen, Nina Rostgaard, Søren Norge Andreassen, Anja Hviid Simonsen, Marianne Juhler, Steen Gregers Hasselbalch, Nanna MacAulay

**Affiliations:** 1 Department of Neuroscience, University of Copenhagen, Blegdamsvej 3B, 2200 Copenhagen, Denmark; 2 Department of Neurosurgery, Rigshospitalet, Copenhagen, Denmark; 3 Danish Dementia Research Centre, Department of Neurology, Rigshospitalet, Copenhagen, Denmark

**Keywords:** Normal pressure hydrocephalus, Cerebrospinal fluid, Inflammatory marker, Biomarkers, Choroid plexus

## Abstract

**Background:**

Idiopathic normal pressure hydrocephalus (iNPH) is a potentially reversible neurological condition of unresolved etiology characterized by a clinical triad of symptoms; gait disturbances, urinary incontinence, and cognitive deterioration. In the present study, we aimed to elucidate the molecular coupling between inflammatory markers and development of iNPH and determine whether inflammation-induced hyperactivity of the choroidal Na^+^/K^+^/2Cl^−^ cotransporter (NKCC1) that is involved in cerebrospinal fluid (CSF) secretion could contribute to the iNPH pathogenesis.

**Methods:**

Lumbar CSF samples from 20 iNPH patients (10 with clinical improvement upon CSF shunting, 10 without clinical improvement) and 20 elderly control subjects were analyzed with the novel proximity extension assay technique for presence of 92 different inflammatory markers. RNA-sequencing was employed to delineate choroidal abundance of the receptors for the inflammatory markers found elevated in the CSF from iNPH patients. The ability of the elevated inflammatory markers to modulate choroidal NKCC1 activity was determined by addition of combinations of rat version of these in ex vivo experiments on rat choroid plexus.

**Results:**

11 inflammatory markers were significantly elevated in the CSF from iNPH patients compared to elderly control subjects: CCL28, CCL23, CCL3, OPG, CXCL1, IL-18, IL-8, OSM, 4E-BP1, CXCL6, and Flt3L. One inflammatory marker, CDCP1, was significantly decreased in iNPH patients compared to control subjects. None of the inflammatory markers differed significantly when comparing iNPH patients with and without clinical improvement upon CSF shunting. All receptors for the elevated inflammatory markers were expressed in the rat and human choroid plexus, except CCR4 and CXCR1, which were absent from the rat choroid plexus. None of the elevated inflammatory markers found in the CSF from iNPH patients modulated the choroidal NKCC1 activity in ex vivo experiments on rat choroid plexus.

**Conclusion:**

The CSF from iNPH patients contains elevated levels of a subset of inflammatory markers. Although the corresponding inflammatory receptors are, in general, expressed in the choroid plexus of rats and humans, their activation did not modulate the NKCC1-mediated fraction of choroidal CSF secretion ex vivo. The molecular mechanisms underlying ventriculomegaly in iNPH, and the possible connection to inflammation, therefore remains to be elucidated.

**Supplementary Information:**

The online version contains supplementary material available at 10.1186/s12987-021-00289-6.

## Background

Idiopathic normal pressure hydrocephalus (iNPH) is a potentially reversible neurological condition first described by Hakim and Adams in 1965 [1]. INPH primarily affects adults above 65 years of age [2, 3] and the condition is characterized by a clinical triad of symptoms; gait disturbances, urinary incontinence, and cognitive deterioration [1, 4]. Patients with iNPH accumulate excessive amounts of cerebrospinal fluid (CSF) in their brain ventricles causing ventricular enlargement [4] and drainage by shunt insertion often improves the patient’s clinical status [5–11]. Such direct reversibility of the clinical symptoms points to excessive CSF accumulation as part of the etiology, rather than permanent brain damage. The intracranial pressure (ICP) in iNPH patients is generally below 15 mmHg [9, 10, 12, 13] which is believed to resemble the ICP in healthy individuals [14, 15], hence the term *normal pressure* hydrocephalus. However, since reference ICP values in healthy individuals remain sparse and pseudo-healthy patients are often used instead [16] it is still debated whether the ICP in this patient group is truly resembling the ICP of healthy individuals. The etiology of iNPH remains unresolved but impaired CSF drainage or CSF hypersecretion may underlie the excessive CSF accumulation and possible ICP elevation. Although no macroscopic obstruction is discernible on diagnostic imaging [4, 17] some iNPH patients display elevated CSF outflow resistance [12, 18, 19] suggesting impaired CSF clearance and absorption. On the other hand, the CSF flow in iNPH patients appears hyperdynamic as demonstrated by an increased aqueductal CSF flow [20–24]. This hyperdynamic CSF flow may originate from a decreased intracranial compliance [13, 25–29] but could alternatively be explained by CSF hypersecretion, a potential mechanism generally neglected in the pathogenesis of hydrocephalus. CSF hypersecretion is sufficient to promote hydrocephalus development in humans [30–32] and animals [33] and has been demonstrated to occur in rats upon activation of an inflammatory pathway involving toll-like receptor 4 and NFκβ in the CSF-producing tissue, the choroid plexus [33]. Altered abundance of select inflammatory markers has been detected in CSF from various hydrocephalic patients [34]. Elevated levels of inflammatory markers may potentially correlate with several factors affecting the prognosis; e.g. the risk of development of some forms of hydrocephalus, the severity of the condition, and the need for surgical intervention [35–38], while treatment with anti-inflammatory drugs reduces the incidence of hydrocephalus and improves clinical outcome [39, 40]. We hypothesize that inflammation-induced CSF hypersecretion may underlie the CSF accumulation seen in iNPH patients, in whom there is no visible CSF drainage obstruction and in whom the underlying pathological mechanism therefore remains unresolved. In the present study, we sought to elucidate the molecular coupling between inflammatory markers and development of iNPH by revealing the inflammatory marker profile in CSF from iNPH patients alongside the delineation of the abundance of the corresponding receptors in the choroid plexus. The connection between the inflammatory marker profile in this patient group and hyperactivity of the choroidal Na^+^/K^+^/2Cl^−^ (NKCC1) transport mechanism, illustrated to sustain approximately 50% of the CSF secretion by the choroid plexus [33, 41, 42], was determined in an ex vivo rodent animal model. If inflammatory markers are involved in the pathogenesis of iNPH, pharmacological targeting of their receptors or downstream signaling pathways could prove a novel treatment strategy.

## Methods

### Study population

Lumbar CSF samples from 20 iNPH patients (mean age: 77 years, range: 71–83 years, 10 M/10 F) and 20 elderly control subjects of similar age and sex distribution (mean age: 67 years, range: 58–81 years, 11 M/9 F) were provided retrospectively by the Danish Dementia Biobank at the Danish Dementia Research Centre (Copenhagen University Hospital, Rigshospitalet, Denmark). The patients were diagnosed in accordance with the international iNPH guidelines [4]; ventricular enlargement on brain imaging, presence of clinical symptoms (gait/balance disturbance present, plus at least one other area of impairment in cognition, urinary symptoms, or both), and normal CSF opening pressure. Gait and incontinence was scored using validated clinical scales [43] and cognition was scored by MMSE [44]. Ten iNPH patients (mean age: 78 years, range: 71–83 years, 5 M/5 F) were classified as “responders” based on improvement of clinical symptoms after surgical insertion of a ventriculo-peritoneal (VP) shunt. The ten remaining iNPH patients (mean age: 76 years, range: 71–82 years, 5 M/5 F) were classified as “non-responders” based on lack of clinical improvement despite surgical intervention with the VP shunt. Response to VP shunt was evaluated 6 months post surgery by reevaluating the clinical symptoms using the clinical scales above. Inclusion criteria for the iNPH group were (1) sample availability from the biobank (2) shunt surgery was performed at the Department of Neurosurgery at Rigshospitalet and (3) follow-up data available on shunt response. All elderly control subjects were referred for diagnostic workup for cognitive impairment including neurological and cognitive examination, diagnostic lumbar puncture, and brain imaging. The clinical investigations did not show any signs of cognitive decline, abnormal CSF, or imaging biomarkers. Furthermore, the subjects did not fulfill the iNPH criteria [4], the criteria for dementia [45], or mild cognitive impairment [46] and were thus included as the elderly control group. Table 1 summarizes the clinical characteristics of the study population. Written informed consent was obtained from patients and control subjects. The study was approved by the Ethics committee of the Capital Region of Denmark.

**Table 1:** Clinical characteristics of the study population

Characteristics	All iNPH patients	iNPH responders	iNPH non-responders	Elderly control subjects
N	20	10	10	20
Age (mean, range)	77y, 71–83y	78y, 71–83y	76y, 71–82y	67y, 58–81y
Sex (M/F)	10/10	5/5	5/5	11/9
MMSE (mean, range)	25 (15–29)	24 (15–29)	26 (23–29)	29 (25–30)
Gait score	3 (1–5)	3 (2–4)	3 (1–5)	NA
Urinary continence score	3 (1–5)	3 (1–5)	3 (1–4)	NA

Gait score: 0—normal, 8—wheelchair bound, Urinary continence score: 0—normal, 6—bladder and bowel incontinence [43]

*iNPH* idiopathic normal pressure hydrocephalus

### Cerebrospinal fluid samples

CSF samples were obtained by lumbar puncture between 9 a.m. and 1 p.m. The first 4 ml were collected for routine investigations and the following 8–12 ml were collected in polypropylene tubes, centrifuged at 2000×*g* for 10 min, the supernatant was aliquoted into cryo tubes and stored at − 80 °C within two hours from sampling according to previous recommendations [47]. The CSF samples were thawed only once for aliquoting prior to analysis. The CSF samples from the iNPH patients were taken as part of the diagnostic workup for iNPH.

### Proximity extension assay

BioXpedia A/S (Aarhus, Denmark) analyzed CSF samples for presence of 92 different inflammatory markers (Inflammation panel, Art. No. 95301) using the novel proximity extension assay (PEA) technique (Olink Bioscience, Sweden) [48]. In brief, the PEA technique employs a pair of oligonucleotide-conjugated antibodies to detect each inflammatory marker in a CSF volume of 1 µl. Upon detection, the oligonucleotides are brought in close proximity, hybridize, and enable DNA polymerization, which produces a PCR sequence. The PCR sequence is amplified and quantified using microfluidic real-time PCR. The PEA technique outputs relative protein values measured in normalized protein expression (NPX), and thus cannot provide the actual concentrations of the markers present in the CSF. The higher the protein abundance in the CSF sample, the higher the NPX value. In addition to the CSF samples, two plasma samples obtained from two randomly chosen elderly control subjects were analyzed for assay validation.

### Experimental animals

All procedures involving experimental animals conformed to the legislations for animal protection and care provided in the European Community Council Directive (2010/63/EU) and complied with the ARRIVE guidelines [49]. Nine-week-old adult male Sprague Dawley rats (Janvier Labs) were used for all experiments. The animals had food and water available ad libitum and were housed with a 12:12 light cycle.

### Choroid plexus isolation and culturing

Rats were anesthetized with an intraperitoneal injection of ketamine and xylazine (60 mg/ml, 6 mg/ml, 0.17 ml per 100 g body weight, ScanVet) and quickly decapitated. Isolated rat brains were immersed in ice-cold artificial CSF (aCSF) containing (in mM): 120 NaCl, 2.5 KCl, 2.5 CaCl_2_, 1.3 MgSO_4_, 1 NaH_2_PO_4_, 10 glucose, 17 Na-HEPES, pH 7.4. Rat brains were kept in ice-cold aCSF for 10 min followed by separation of the two hemispheres and isolation of the choroid plexus from the lateral ventricles. The acutely isolated lateral choroid plexus was placed in Dulbecco’s Modified Eagle’s Medium **(**DMEM1965, high glucose, NaHCO_3_^−^, HEPES, Gibco) supplemented with penicillin–streptomycin (100 U/ml, 100 µg/ml, Gibco), rat epidermal growth factor (10 ng/ml, SRP3238, Sigma), and fetal bovine serum (10%, 04-007-1A, Biological Industries) (DMEM growth medium). As inflammatory markers may exert their effect on cells and tissue on a longer time scale, the choroid plexus was incubated for 16 h at 37 °C, 5% CO_2,_ in presence of select inflammatory markers (or vehicle). To verify choroid plexus viability during the 16 h culturing ex vivo, we performed calcein acetoxymethyl (calcein-AM) staining and evaluated the tissue by microscopy [50]. Viable cells contain active intracellular esterases that cleave the AM group from the calcein, resulting in a bright fluorescent signal from these viable cells. After isolation from the lateral ventricles, the choroid plexus tissue was placed in DMEM growth medium and left for 16 h at 37 °C, 5% CO_2_ prior to incubation in aCSF containing calcein-AM (16.7 µM) for 10 min at room temperature. An acutely isolated choroid plexus was employed as a positive control and the negative control consisted of an acutely isolated choroid plexus placed in distilled water (dH_2_O) for 16 h prior to calcein-AM staining. Images were acquired using Zeiss Axioplan 2 microscope equipped with epifluorescence and interference filters with a 702 moni AxioCam, and using Zeiss Zen Black software.

### Isotope efflux experiments

Following 16 h of incubation, the choroid plexus was allowed to recover for 10 min in 37 °C aCSF prior to 10 min of incubation in isotope solution containing: ^86^Rb^+^ (1 µCi/ml, 022-105721-00321-0001, POLATOM) and ^3^H-mannitol (4 µCi/ml, NET101, Perkin Elmer). ^3^H-mannitol does not enter the choroid plexus and serves as an extracellular marker [51]. After isotope incubation, the choroid plexus was briefly rinsed in isotope-free aCSF followed by transfer into new wells containing isotope-free aCSF at regular intervals as indicated on the figure. For every time point, 0.2 ml of surrounding aCSF was collected and placed into scintillation vials. At the end of the experiment, the choroid plexus was placed into a scintillation vial containing 0.2 ml Solvable (6NE9100, Perkin Elmer) and dissolved at room temperature for a minimum of 4 h. The isotope content was determined in 2 ml Ultima Gold™ XR scintillation liquid (6013119, Perkin Elmer) using the Tri-Carb 2900TR Liquid Scintillation Analyzer (Packard). For each time point, the ^86^Rb^+^ activity was corrected for extracellular background using ^3^H-mannitol. Data are shown as the natural logarithm of the ^86^Rb^+^ activity at time each point (A_T_) normalized to the initial ^86^Rb^+^ activity (A_0_) as a function of time. The slope from linear regression analysis was used to determine the ^86^Rb^+^ efflux rate constant (min^−1^) in absence (including vehicle) and presence of bumetanide (B3023, Sigma; 20 µM made up from a stock solution of 20 mM in DMSO) or select inflammatory markers [41, 51].

### RNA sequencing

STAR-RNA and RSEM parameter settings for the library build, mapping and quantification, together with all scripts for the gene annotation and analysis are available online at https://github.com/Sorennorge/MacAulayLab-RNAseq1 [52]. Rat choroid plexus (laterals and 4th) was isolated in ice-cold aCSF and stored in RNAlater (R0901, Sigma) at − 80 °C. The RNA extraction and library preparation were performed by Novogene Company Limited, UK with NEB Next^®^ Ultra™ RNA Library Prep Kit (NEB, USA) prior to RNA sequencing (paired-end 150 bp, with 15 Gb output) on an Illumina NovaSeq 6000 (Illumina, USA). Novogene performed QC and removed low quality reads and adapters. The 150 base paired-end reads were mapped to Reference genome Rnor_6.0 (Rattus_norvegicus) using Spliced Transcripts Alignment to a Reference (STAR) RNA-seq aligner (v 2.7.2a) [53]. The mapped alignment generated by STAR was normalized to transcripts per million (TPM) [54] with RSEM (v. 1.3.3) [55]. Gene information was gathered with mygene (v 3.1.0) python library [56, 57], https://mygene.info [58]. The human choroid plexus data was publicly available and obtained from the Gene Expression Omnibus (GEO) database, accession number GSE137619 (SRR10134643-SRR10134648), https://www.ncbi.nlm.nih.gov/geo/query/acc.cgi?acc=GSE137619 [59]. The data were quality controlled with Fastqc (available online at http://www.bioinformatics.babraham.ac.uk/projects/fastqc [60]) and trimmed with Trimmomatic [61] (quality below 20, minimum length of 35 bp). The six human choroid plexus samples were mapped, with similar settings as the rat choroid plexus sample, to the reference genome GRCh38 (Homo sapiens) with STAR and quantified with RSEM, and the mean TPM of the six samples was used for the analysis.

### Inflammatory markers for ^86^Rb^+^ efflux experiments

Rat interleukin-18 (IL-18, cat. no. 521-RL-025) was purchased from R&D Systems (UK). Rat C–C motif chemokine ligand 28 (CCL28, cat. No. chm-278), rat CCL6 (cat. no. chm-268), rat CCL3 (cat. no. chm-343), and rat oncostatin-M (OSM, cat. no. cyt-169) were purchased from ProSpec (Israel). Rat C-X-C motif chemokine ligand 1 (CXCL1, Cat. No. SRP3240) was purchased from Sigma (USA). All chemicals were dissolved in sterile water or phosphate buffered saline supplemented with 0.1% bovine serum albumin (A6003, Sigma) to a stock concentration of 100 µg/ml and kept frozen in aliquots at − 20 °C until use to avoid repeated freeze–thaw cycles. Final concentrations are indicated in the Figure legends.

### Statistics

Data analysis and statistical tests were conducted in GraphPad Prism version 8 (GraphPad Software). *Inflammatory markers in CSF:* The limit of detection (LOD) was calculated for each inflammatory marker as the background signal plus two times the standard deviation of the inflammatory marker. Inflammatory markers were excluded from further analysis if > 35% of the samples were below the LOD. This criterion led to the exclusion of 33 inflammatory markers. None of the excluded inflammatory markers showed any tendency towards preferential presence in one of the study groups. The remaining 59 inflammatory markers were included in the statistical analysis. Additional file 1: Table S1, provides an overview of the excluded and included inflammatory markers. For normally distributed data, an unpaired two-tailed T-test was conducted and a Welch’s correction was applied if variances were unequal. For non-normally distributed data, a Mann–Whitney test was conducted. P < 0.05 was considered statistically significant. P-values were not corrected for multiple comparisons [62] as it was desired to identify as many potential candidates as possible for subsequent rodent functional experimentation, which would determine the pathophysiological role of the inflammatory markers on choroidal CSF secretion. Additional file 2: Table S2 and Additional file 3: Table S3, provide an overview of the statistical tests and results for each inflammatory marker. ^*86*^*Rb*^+^ *efflux experiments:* Data are presented as mean ± standard deviation and n corresponds to the number of individual animals. Data were analyzed with an unpaired two-tailed t-test and P < 0.05 was considered statistically significant.

## Results

### Inflammatory markers are elevated in CSF from iNPH patients

To determine if iNPH patients displayed altered CSF distribution of inflammatory markers, we compared lumbar CSF from 20 iNPH patients and 20 elderly control subjects. The clinical characteristics of the study groups are summarized in Table 1. Quantification of 92 different inflammatory markers was obtained using the novel PEA technique, which relies on dual antibody-based recognition of each inflammatory marker, providing a high throughput with excellent specificity and sensitivity [48]. The CSF levels of 11 inflammatory markers were significantly elevated in iNPH patients compared to elderly control subjects: CCL28, CCL23, CCL3, osteoprotegerin (OPG), CXCL1, IL-18, IL-8, OSM, eukaryotic translation initiation factor 4E-binding protein 1 (4E-BP1), CXCL6, and fms-related tyrosine kinase 3 ligand (Flt3L) (Table 2). The majority of the elevated inflammatory markers were chemokines (CCL28, CCL23, CCL3, CXCL1, and CXCL6) or interleukins (IL-18 and IL-8), small secreted proteins that drive and modulate immune responses in a pleiotropic manner, depending on their target [63, 64].

**Table 2:** Elevated inflammatory markers in CSF from iNPH patients

Inflammatory marker	Full name	iNPH patients	Elderly control subjects	Percent increase (%)	P-value
CCL28	C–C motif chemokine ligand 28	1.749 ± 0.222	1.495 ± 0.265	19	0.0007
CCL23	C–C motif chemokine ligand 23	3.260 ± 0.451	2.993 ± 1.013	20	0.0051
CCL3	C–C motif chemokine ligand 3	4.506 ± 0.433	4.262 ± 0.973	18	0.0056
OPG	Osteoprotegerin	10.150 ± 0.529	9.765 ± 0.478	31	0.0207
CXCL1	C-X-C motif chemokine ligand 1	6.646 ± 0.524	6.408 ± 1.091	18	0.0211
IL-18	Interleukin-18	2.639 ± 0.595	2.409 ± 1.093	17	0.0227
IL-8	Interleukin-8	8.497 ± 0.506	8.275 ± 0.787	17	0.0263
OSM	Oncostatin-M	0.726 ± 0.373	0.655 ± 0.915	5	0.0283
4E-BP1	Eukaryotic translation initiation factor 4E-binding protein 1	2.763 ± 0.653	2.544 ± 1.177	16	0.0283
CXCL6	C-X-C motif chemokine ligand 6	4.246 ± 0.637	3.929 ± 1.353	25	0.0375
Flt3L	Fms-related tyrosine kinase 3 ligand	9.100 ± 0.356	8.909 ± 0.515	14	0.0402

Data are expressed as normalized protein expression (NPX) values (mean ± SD), an arbitrary unit on log_2_ scale. Percent increase represents the increase in iNPH CSF compared to CSF from elderly control subjects. Data were analyzed with an unpaired two-tailed T-test or a Mann–Whitney test. iNPH = idiopathic normal pressure hydrocephalus

The CSF level of one inflammatory marker, CUB domain-containing protein 1 (CDCP1), a transmembrane glycoprotein chemotactic for T-cells [65], was significantly decreased in iNPH patients compared to the group of elderly control subjects (iNPH: 4.576 ± 0.245 NPX; elderly control subjects: 4.905 ± 0.556 NPX, P = 0.0225).

INPH patients often improve clinically upon VP shunt insertion [5–11]. However, as some patients fail to improve, the underlying etiology may potentially differ, which could give rise to differences in the CSF distribution of inflammatory markers. To determine if the inflammatory profile of iNPH patients with clinical improvement upon VP shunting (iNPH responders) differed from those who did not improve (iNPH non-responders), we compared the inflammatory CSF marker content of the two patient groups, but found none of the inflammatory markers to differ significantly (see Additional file 2: Table S2 and Additional file 3: Table S3, for all statistical tests). Taken together, our findings demonstrate that the molecular profile of the CSF from iNPH patients contains elevated levels of inflammatory markers and that the inflammatory marker profile of iNPH responders and iNPH non-responders is indistinguishable prior to VP shunt insertion.

### Inflammatory receptors are present in the choroid plexus

Of the 11 inflammatory markers significantly elevated in the CSF from iNPH patients, eight were selected as candidates for further ex vivo investigation of their ability to modulate the transport activity in choroid plexus: CCL28, CCL23, CCL3, CXCL1, IL-18, IL-8, OSM and CXCL6. The remaining inflammatory markers OPG, 4E-BP1, and Flt3L were not investigated further as they serve physiological roles which make them less likely to directly influence choroidal CSF secretion.

**Fig. 1:**
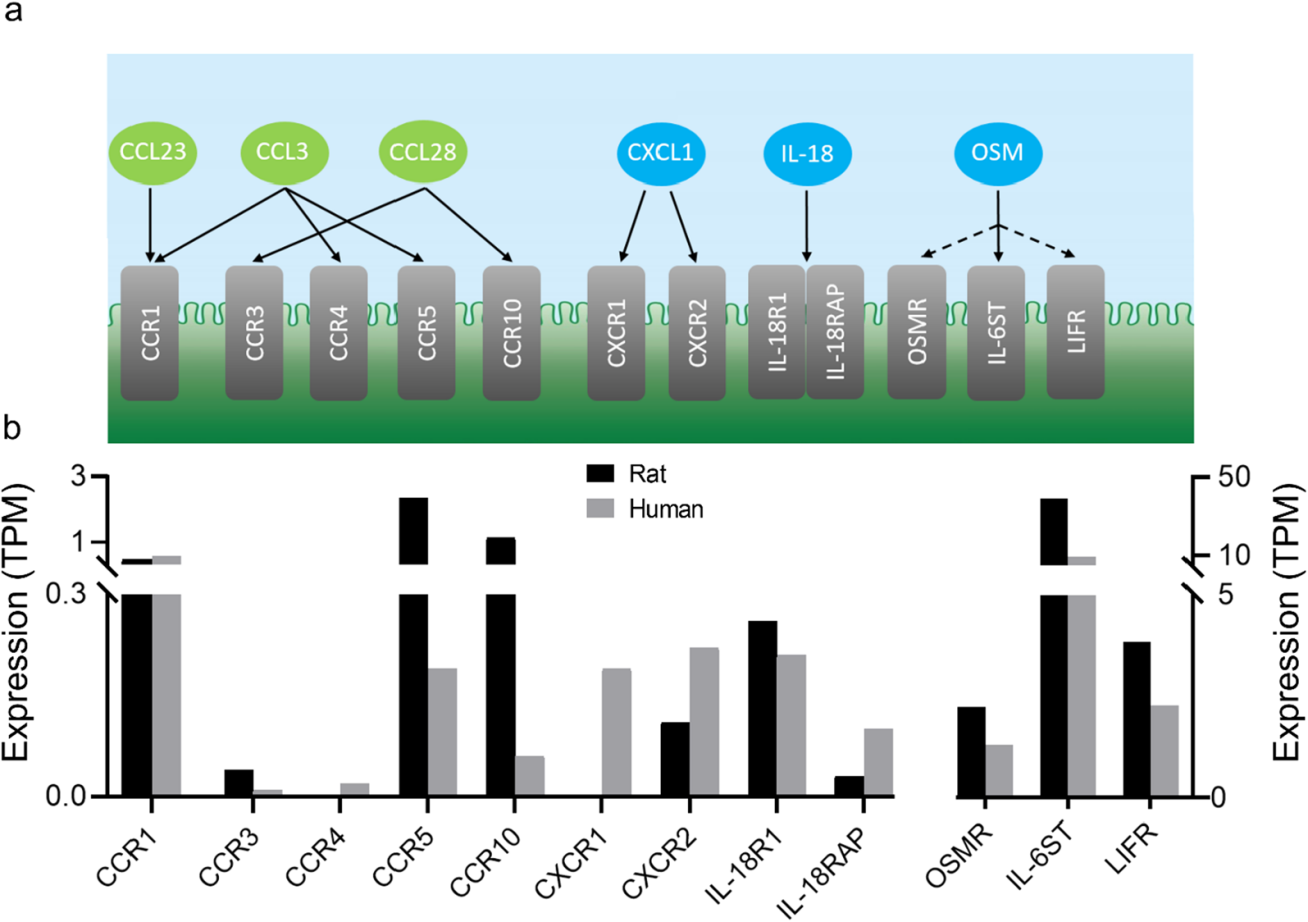
Inflammatory receptors are present in the choroid plexus. **a** Schematic illustration of the inflammatory markers and their corresponding receptors. The inflammatory markers (green and blue circles) and their corresponding receptors (grey bars) are indicated by the black arrows. For OSM, the dotted black arrows indicate binding to IL-6ST in combination with either OSMR or LIFR. The illustration is based on the information provided in [67, 88, 102, 106]. **b** Expression of the inflammatory receptors in the rat and human choroid plexus. The expression levels were evaluated with RNA-seq and given in transcript per million (TPM). The human choroid plexus data was obtained from the GEO database, accession number GSE137619 [59]

The ability of the eight candidate inflammatory markers to alter choroidal transporter activity requires that their respective receptors are expressed in choroid plexus (Fig. 1a). Such verification was obtained by transcriptional analysis (RNA-seq) of the choroid plexus. The ex vivo determination of choroid plexus transport activity cannot be conducted in human choroid plexus. Therefore we employed rat choroid plexus for the functional assay described below and hence performed the RNA-seq on acutely isolated rat choroid plexus. RNA-seq revealed that the majority of the inflammatory receptors targeted by the detected inflammatory markers were expressed in the rat choroid plexus, albeit at variable levels (Fig. 1b). The inflammatory receptors C–C motif chemokine receptor 1 (CCR1), CCR3, C-X-C motif chemokine receptor 2 (CXCR2), IL-18 receptor 1 (IL-18R1), and IL-18 receptor accessory protein (IL-18RAP) were expressed at low levels (transcript per million (TPM) < 1), CCR5, CCR10, OSM receptor (OSMR), and leukemia inhibitory factor receptor (LIFR) at intermediate levels (1–5 TPM), and IL-6 signal transducer (IL-6ST) at high level (TPM = 39). Two inflammatory receptors targeted by the elevated inflammatory agents, CCR4 and CXCR1, were absent from the rat choroid plexus. To determine whether the rat choroid plexus expression levels were representative of that of humans, we compared our RNA-seq data with previously published RNA-seq choroid plexus data from humans (n = 6, age: 62 ± 6.1 y, 2 M/4 F, GEO database accession number GSE137619, https://www.ncbi.nlm.nih.gov/geo/query/acc.cgi?acc=GSE137619 [59], Fig. 1b). All inflammatory receptors targeted by the elevated inflammatory markers were expressed in the human choroid plexus. Hence, the only noticeable differences between humans and rats were absence of CCR4 and CXCR1 in the rat choroid plexus. Taken together, these data demonstrate choroidal expression of inflammatory receptors being targeted by the inflammatory markers found elevated in iNPH patients and thus support the notion that inflammatory markers can act directly on the choroid plexus and possibly modulate its functional properties.

### Inflammatory markers do not modulate NKCC1 activity in rat choroid plexus

**Fig. 2:**
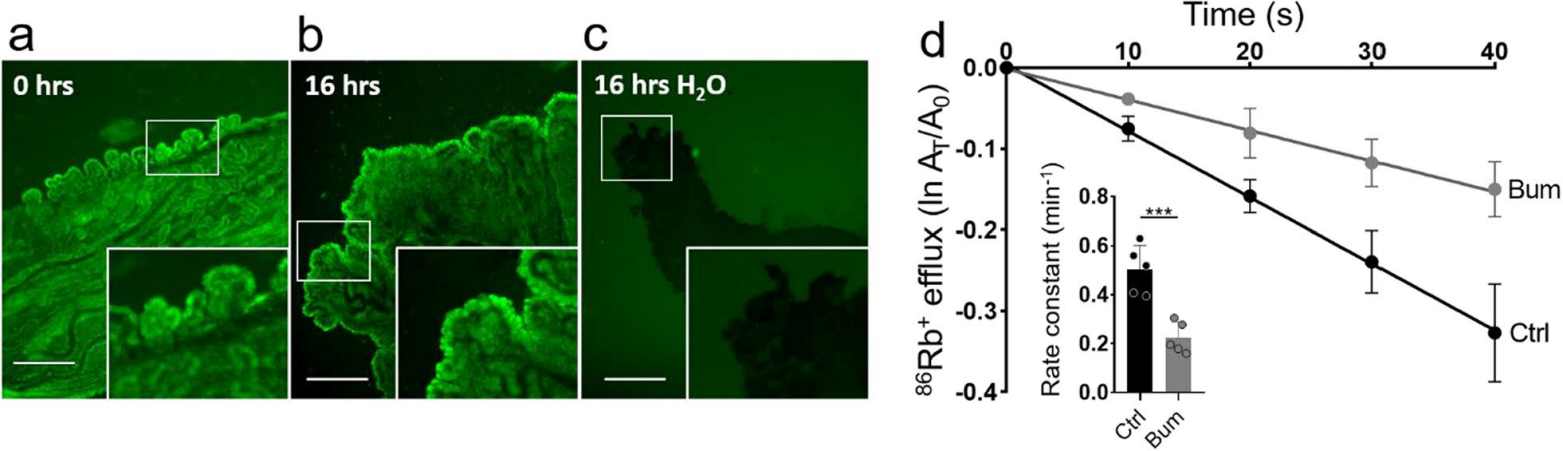
The choroid plexus remains viable ex vivo. **a** Calcein-AM staining of choroid plexus directly after isolation (0 h). **b** Calcein-AM staining of choroid plexus after 16 h of incubation in cell culture medium (16 h). **c** Calcein-AM staining of choroid plexus after 16 h of incubation in sterile water (16 h H_2_O), scale bars 500 μm. Inserts in **a-c** contain representative regions of the choroid plexus (white boxes) upon ×5 magnification. **d** ^86^Rb^+^ efflux from the choroid plexus as a function of time in control solution (ctrl, n = 5) or in presence of 20 µM bumetanide (bum, n = 5). The y-axis represents the natural logarithm of the ^86^Rb^+^ amount left in the choroid plexus at time T (A_T_) divided by the initial amount at time 0 (A_0_). Insert: ^86^Rb^+^ efflux rate constant in control solution (0.50 ± 0.10 min^−1^, n = 5) or in presence of 20 µM bumetanide (0.22 ± 0.06 min^−1^, n = 5), P = 0.0008. Error bars in **d** represent standard deviation and statistical significance was tested with an unpaired two-tailed t-test. ***P < 0.001

To estimate the influence of the inflammatory markers that were elevated in iNPH patients on choroidal CSF secretion, we determined their effect on the activity of the choroidal water-transporting Na^+^/K^+^/2Cl^−^ cotransporter NKCC1, a key contributor to choroidal CSF secretion [33, 41, 42]. To this end, we employed ex vivo rat choroid plexus exposed to the rat version of the respective inflammatory markers. As inflammatory markers may exert their effects on a longer time scale, the choroid plexus was placed in cell culture medium and incubated for 16 h in presence of the inflammatory markers. Each epithelial cell of the choroidal monolayer was thus directly in touch with the oxygenated and nutrient-rich culture medium, like individual cells in culture. The viability of the choroid plexus epithelial cells was confirmed with a calcein-AM survival assay (Fig. 2a–c), which demonstrated that the epithelial cells were viable at the end of the culturing period. The transport activity of NKCC1 in the 16 h cultured ex vivo choroid plexus was assessed by rubidium (^86^Rb^+^) efflux experiments, in which ^86^Rb^+^ replaces K^+^ at its binding site and acts as a tracer for the K^+^ transport through NKCC1. Inclusion of the NKCC1-specific inhibitor bumetanide (20 µM) diminished the choroidal ^86^Rb^+^ efflux by ~ 56% (Fig. 2d, n = 5, P = 0.0008), thus demonstrating that a large fraction of the ^86^Rb^+^ efflux occurred via NKCC1 in this experimental set-up. The ^86^Rb^+^ assay thus allows for direct assessment of the effect of the inflammatory markers on the choroidal NKCC1 activity.

**Fig. 3:**
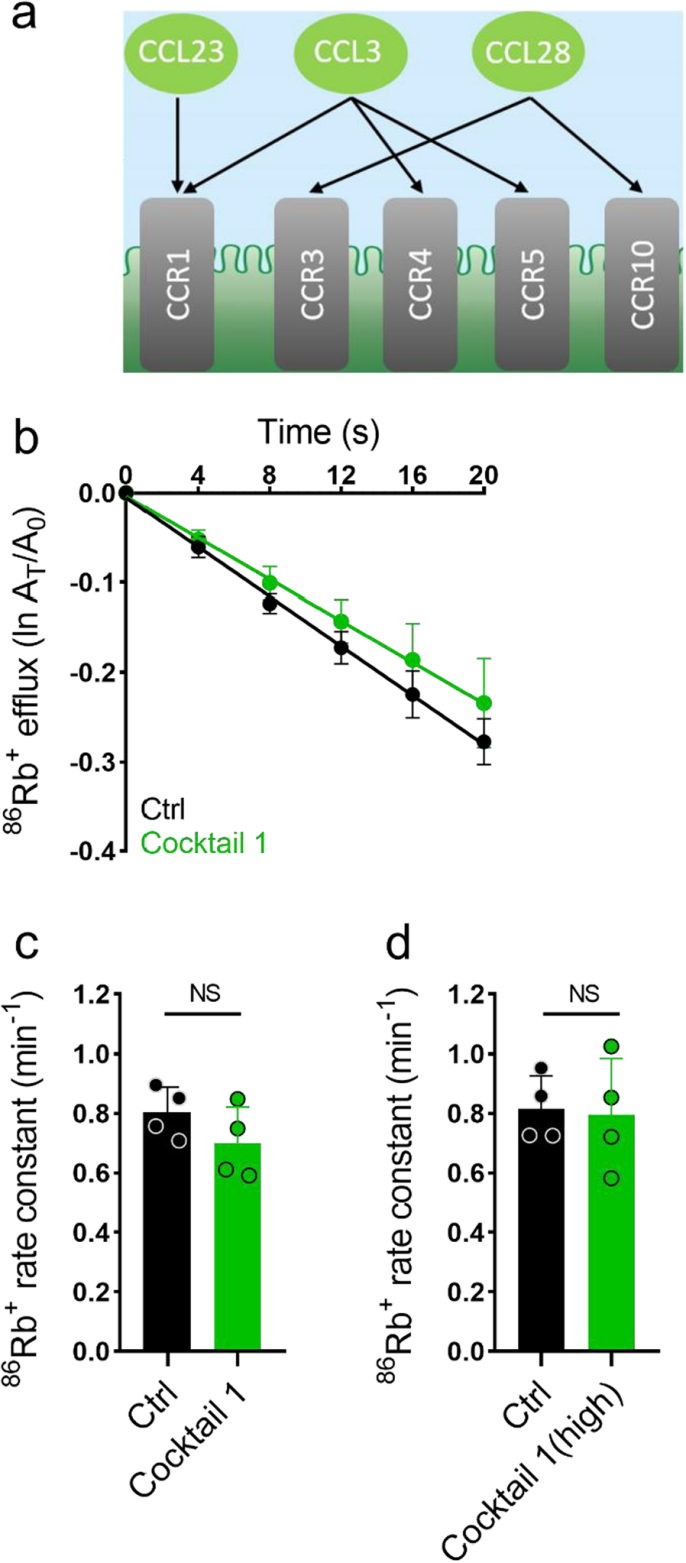
The inflammatory markers CCL28, CCL23, and CCL3 do not modulate the NKCC1 activity. **a** Schematic illustration of the inflammatory markers in cocktail 1 (CCL28, CCL23, and CCL3) and their corresponding receptors. **b** ^86^Rb^+^ efflux from the rat choroid plexus as a function of time in the control setting (black, n = 4) or with exposure to cocktail 1 (green, n = 4) containing CCL28 (100 ng/ml), CCL23 (200 ng/ml), and CCL3 (100 ng/ml). The y-axis represents the natural logarithm of the ^86^Rb^+^ amount left in the choroid plexus at time T (A_T_) divided by the initial amount at time 0 (A_0_). **c** ^86^Rb^+^ efflux rate constant in the control setting (0.80 ± 0.09 min^−1^, n = 4) or with exposure to cocktail 1 (0.70 ± 0.12 min^−1^, n = 4, CCL28 (100 ng/ml), CCL23 (200 ng/ml), and CCL3 (100 ng/ml). P = 0.21. **d** ^86^Rb^+^ efflux rate constant in the control setting (0.82 ± 0.11 min^−1^, n = 4) or with exposure to high concentrations of cocktail 1 (0.80 ± 0.19 min^−1^, n = 4, CCL28 (500 ng/ml), CCL23 (500 ng/ml), and CCL3 (500 ng/ml), P = 0.86. Error bars in **b**, **c** and **d** represent standard deviation and statistical significance was tested with an unpaired two-tailed t-test. NS, not significant

**Fig. 4:**
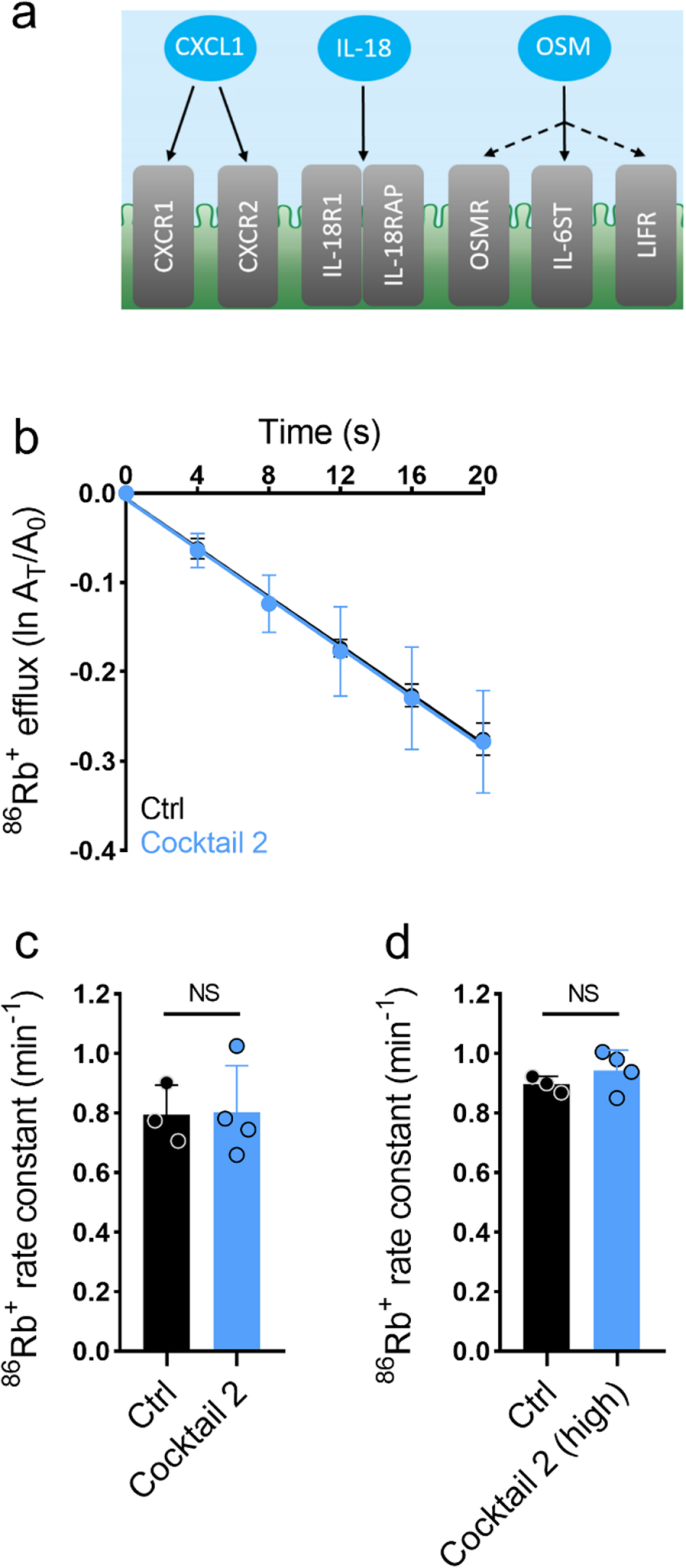
The inflammatory markers IL-18, CXCL1, and OSM do not modulate the NKCC1 activity. **a** Schematic illustration of the inflammatory markers in cocktail 2 (IL-18, CXCL1, and OSM) and their corresponding receptors. **b** ^86^Rb^+^ efflux from the rat choroid plexus as a function of time in the control setting (black, n = 3) or with exposure to cocktail 1 (blue, n = 4) containing IL-18 (200 ng/ml), CXCL1 (100 ng/ml), and OSM (200 ng/ml). The y-axis represents the natural logarithm of the ^86^Rb^+^ amount left in the choroid plexus at time T (A_T_) divided by the initial amount at time 0 (A_0_). **c** ^86^Rb^+^ efflux rate constant in the control setting (0.79 ± 0.10 min^−1^, n = 3) or with exposure to cocktail 2 (0.80 ± 0.16 min^−1^, n = 4) containing IL-18 (200 ng/ml), CXCL1 (100 ng/ml), and OSM (200 ng/ml). P = 0.94. **d** ^86^Rb^+^ efflux rate constant in the control setting (0.90 ± 0.03 min^−1^, n = 3) or with exposure to high concentrations of cocktail 2 (0.94 ± 0.07 min^−1^, n = 4, IL-18 (500 ng/ml), CXCL1 (500 ng/ml), and OSM (500 ng/ml)), P = 0.32. Error bars in **b**–**d** represent standard deviation and statistical significance was tested with an unpaired two-tailed t-test. NS, not significant

The elevated inflammatory markers were grouped into two cocktails to determine their effect on NKCC1. Cocktail 1 contained the most significantly elevated inflammatory markers in the iNPH patient samples (CCL28, CCL23, CCL3; P < 0.001, Fig. 3a). As rat CCL6 is orthologous to human CCL23 [66], rat CCL6 was employed to mimic human CCL23 signaling. The ^86^Rb^+^ efflux rate (read-out of NKCC1 transport activity) from the rat choroid plexus was not significantly altered by exposure to cocktail 1 (Fig. 3b, c, n = 4, P = 0.21), nor to excessively high concentrations of these (Fig. 3d, n = 4, P = 0.86). Cocktail 2 (IL-18, CXCL1, and OSM) targeted the remaining inflammatory receptors (Fig. 4a). Since CXCL1, CXCL2, and IL-8 activate the same receptors, CXCR1 and CXCR2 [67] (Fig. 1a), only one inflammatory marker, CXCL1, was chosen to activate these. Application of cocktail 2 (IL-18, CXCL1, and OSM) did not alter the ^86^Rb^+^ efflux rate either (Fig. 4b, c, n = 3–4, P = 0.94), nor did the excessively high concentrations of these (Fig. 4d, n = 3–4, P = 0.32). As our RNA-seq analysis revealed absence of CCR4 and CXCR1 from the rat choroid plexus, signaling through these receptors was not investigated. Hence, none of the combinations of inflammatory markers found in the CSF from iNPH patients modulated the rat choroidal NKCC1 activity. Taken together, our findings demonstrate that although inflammatory markers are present at elevated levels in the CSF from iNPH patients, they do not directly alter the fraction of the choroidal CSF secretion predicted to be mediated by NKCC1 transport activity on the tested time scale.

## Discussion

This study, based on the novel PEA technique, uncovers the inflammatory profile of the CSF from iNPH patients investigating the largest number of inflammatory markers so far and providing novel insight into the inflammatory marker profile of CSF from iNPH patients. We here demonstrate that the CSF from iNPH patients contains elevated levels of a subset of inflammatory markers. The corresponding inflammatory receptors are, in general, expressed in the choroid plexus of rats and humans, but their activation did not stimulate NKCC1 that is highly expressed in the luminal membrane of choroid plexus in mice, rats, and humans [41, 68, 69] and demonstrated to support approximately half of the CSF secreted in the tested species (mice, rats, dogs; [33, 41, 42]). Note that the NKCC1 inhibitor bumetanide only exerts its inhibitory action on NKCC1-mediated CSF secretion when delivered directly into the cerebral ventricles of the experimental animal (to target NKCC1 at the luminal membrane of choroid plexus [33, 41, 42]) and not upon administration of bumetanide i.v. or i.p., in which cases, the inhibitor fails to reach its choroidal target [70, 71]. Inflammatory markers therefore appear *not* to promote iNPH via induction of choroidal CSF hypersecretion by promotion of NKCC1 activation, at least not in the rat ex vivo model here employed. Based on the present data set, however, we cannot rule out that other transport mechanisms involved in CSF secretion (see [71] for review) could be modulated by these inflammatory agents.

Eleven inflammatory markers were elevated in CSF obtained from iNPH patients compared to elderly control subjects. Three inflammatory markers (OPG, 4E-BP1, and Flt3L) were not investigated experimentally, as they were unlikely to directly influence choroidal CSF secretion: OPG acts as a decoy receptor [72], 4E-BP1 represses translation and plays a predominant role in cell proliferation [73], while Flt3L, a hematopoietic growth factor, promotes differentiation of hematopoietic stem cells, particularly dendritic cells [74]. The remaining eight inflammatory markers were investigated for their ability to alter choroidal CSF secretion: CCL28, CCL23, CCL3, CXCL1, IL-18, IL-8, OSM and CXCL6.

CCL28 is a chemokine constitutively expressed by epithelial cells at mucosal sites [75], which promotes chemotaxis of immune cells through the receptors CCR3 and CCR10 [75, 76] and is inducible upon presence of inflammatory or infectious stimuli [77]. CCL28 is elevated in CSF from patients with Parkinson’s disease (PD) [78], who have normal CSF production [79], and its presence in iNPH patients may instead indicate similar pathological features of neurodegeneration and/or inflammation [80]. The chemokine CCL23 is expressed mainly by macrophages and exerts its chemotactic effects on immune cells through CCR1 [81, 82]. Elevated CCL23 levels are reported in various inflammatory diseases [83, 84] and is, in addition, associated with progression from mild cognitive impairment to Alzheimer’s disease (AD) [85]. Elevated CCL23 in both AD and iNPH aligns well with the overlapping clinical characteristics of iNPH and AD [86] and the concomitant AD pathology found in iNPH [87]. CCL3 is a chemokine that exerts its proinflammatory actions through the receptors CCR1 and CCR5, and with less affinity through CCR4 [88]. CCL3 is secreted by various cells such as monocytes, macrophages, and epithelial cells [89] and is inducible by proinflammatory agents such as lipopolysaccharides, tumor necrosis factor α, and IL-1β [90, 91]. CCL3 is elevated in CSF from patients with posthemorrhagic hydrocephalus [92] and hydrocephalus associated with tuberculous meningitis [93], which both associate with inflammation [94]. Elevated CCL3 in iNPH patients suggests similar inflammatory conditions in this patient group.

The chemokines CXCL1, CXCL6, and IL-8 exert their actions through the receptors CXCR1 and CXCR2 [67]. CXCL1 is elevated in brain tissue from hydrocephalic mice with genetically induced ciliary dysfunction, in which hydrocephalus develops concurrent with neuroinflammation and tissue injury [95]. As no antecedent proinflammatory insult occurs in these mice, the authors conclude that neuroinflammation arises as a consequence of the intracranial changes associated with hydrocephalus development [95]. Elevated CXCL1 in iNPH patients may therefore reflect presence of neuroinflammation and it may arise concomitantly with development of hydrocephalus. Like CXCL1, CXCL6 serves as a neutrophil chemoattractant [96] and is elevated in bacterial and viral meningitis [97].

Several studies have investigated the proinflammatory chemokine IL-8 in relation to hydrocephalus. While some studies report elevated IL-8 in CSF from patients with different hydrocephalic conditions [93, 98, 99], others report unaltered levels [92, 98–101]. Our findings of elevated IL-8 in CSF from iNPH patients are in agreement with the findings by Czubowicz [98] but contradict others [99–101]. Although the contradicting findings may result from methodological differences, additional studies are required to determine whether elevated CSF levels of IL-8 are characteristic of iNPH. IL-18 is a proinflammatory cytokine released in response to inflammatory and infectious stimuli which signals through the receptors IL-18R1 and IL-18RAP and is involved in various neurological conditions [102]. IL-18 is elevated in CSF from preterm infants with posthemorrhagic hydrocephalus and hydrocephalus associated with spina bifida and aqueductal stenosis [103, 104]. As these conditions are associated with inflammatory changes including astrogliosis, microgliosis, and white matter damage [94, 103–105], it can be speculated whether similar changes occur in iNPH patients.

OSM is a pleiotropic interleukin-6-type cytokine that utilizes two receptor complexes, IL-6ST/LIFR and IL-6ST/OSMR, to mediate a broad range of homeostatic activities, some of which include hematopoiesis, liver regeneration, and homeostasis of neural precursor cells [106, 107]. Overproduction of OSM is associated with skin and airway inflammation, inflammatory bowel disease, and other pathological conditions [106, 108, 109]. OSM was recently identified as a potential CSF marker of general CNS inflammation, although interestingly, the study included iNPH patients and patients with idiopathic intracranial hypertension as the non-inflamed controls [110]. The elevated OSM levels in CSF from iNPH patients in the present study are indicative of inflammation, but the inflammatory condition may be less pronounced in comparison to other patient groups with prominent signs of inflammation such as those suffering from meningitis or subarachnoid hemorrhage [110].

One inflammatory marker, CDCP1, was significantly decreased in the CSF obtained from iNPH patients compared to elderly control subjects. CDCP1 is a transmembrane glycoprotein chemotactic for T-cells [65] that has been associated with cell adhesion and cancer development [111]. Although its physiological role in iNPH patients remains unclear, it can be speculated whether CDCP1 may serve as a diagnostic marker, potentially distinguishing iNPH from other types of hydrocephalus.

Inflammatory markers such as cytokines, interleukins, and chemokines signal through various intracellular pathways and are capable of acting synergistically or antagonistically [112]. In the present study, the elevated inflammatory markers were combined into cocktails and the ability of the inflammatory markers to modulate choroidal CSF secretion was thus not assessed individually. The combination of the inflammatory markers into cocktails may have compromised the effect of each inflammatory marker. However, as the inflammatory markers were elevated collectively in the CSF from iNPH patients, we sought to mimic the nature of iNPH by combining the inflammatory markers. In the present study, we did not obtain evidence that inflammatory markers can act directly on the choroid plexus to promote such hyperactivation of NKCC1, as was observed in an in vivo rodent model of intraventricular hemorrhage [33]. The excised choroid plexuses were kept in tissue culture conditions for 16 h to allow the inflammatory agents to serve their purpose via modulation of transcriptional and translational processes, including post-translational modifications such as phosphorylation of proteins, which has been described earlier for NKCC1-mediated CSF hypersecretion [33]. However, it is possible that all elevated inflammatory markers have to be included in the same experiment, that additional time is required for the inflammatory markers to have an effect on the choroidal transport protein NKCC1 (and/or target other choroidal transporters), or that lesser time is required for optimal initiation of the inflammatory machinery: Other studies have shown that upon an inflammatory stimulus, the choroid plexus transcriptome responds rapidly within 3–6 h [113, 114] followed by gradual return to baseline within 72 h [113]. However, whereas these studies only applied an inflammatory stimulus once, we exposed the choroid plexus to the inflammatory markers continuously for 16 h and would therefore not expect the effects of the inflammatory markers on the choroidal CSF secretion to subside. Although we document choroid plexus epithelial cell viability and bumetanide-sensitive ^86^Rb^+^ efflux following the 16 h incubation time, prolonged presence of the inflammatory markers may have initiated oxidative stress, apoptosis, or other cellular mechanisms that could have compromised potential effects of the inflammatory markers on choroidal NKCC1-mediated ion transport. However, other research groups have reported successful exposure of choroid plexus epithelial cell lines to inflammatory stimuli for longer time periods [115].

The inability of the inflammatory markers to modulate choroidal NKCC1 transport activity ex vivo and thus potentially contribute to development of iNPH leaves the molecular coupling between inflammatory markers and iNPH unresolved. Elevated levels of inflammatory markers in CSF from iNPH patients indicate presence of an ongoing inflammatory condition. However, whether this inflammatory condition promotes iNPH development or arises secondarily to the pathological changes observed in the iNPH brain remains uncertain. Some studies demonstrate that CSF levels of inflammatory markers decrease following CSF shunting in parallel with clinical improvement [116, 117] suggesting that the inflammatory condition is intrinsically related to disturbed CSF dynamics. Interestingly, we did not find any significant differences in the CSF distribution of inflammatory markers when comparing iNPH shunt responders and iNPH shunt non-responders. Our findings therefore illustrate that the inflammatory profile of iNPH patient CSF obtained during the diagnostic workup cannot be used to predict the outcome of VP shunting in this patient group. One must, however, bear in mind that shunt response may be affected by other factors not accounted for in the present study, including clinical complications and comorbidities. In the future, studies should aim to resolve the role of inflammation in the pathogenesis of iNPH and thereby elucidate whether alleviation of the inflammation condition may prove beneficial in treatment of iNPH.

The present study is associated with certain limitations. Although all included iNPH patients were in moderate to severe stage of disease (see “Methods”), the exact time between onset of symptoms and time of CSF sampling is unknown and could affect the inflammatory profile of the patients. The elderly control group is of an average age of 10 years below that of the iNPH patients. Since inflammation tends to increase with age, such age difference could affect the obtained results. We quantified the inflammatory marker content in CSF from human iNPH patients sampled at one time point only. Since an animal model of iNPH does not currently exist, the ability of (the rat version of) these elevated inflammatory markers to modulate choroidal NKCC1 activity was investigated in rat choroid plexus, as these experiments would not be possible with human tissue. Although our RNA-seq analysis revealed that the majority of the corresponding inflammatory receptors were expressed in the choroid plexus of rats and humans, we cannot exclude that differences between species (humans and rats) may have compromised the findings of our study. Direct modulation of choroidal CSF secretion requires that the receptors for the elevated inflammatory markers are present on the CSF-facing membrane of the choroid plexus. As our RNA-seq analysis only allowed for determination of choroidal receptor expression, not membrane localization, we cannot exclude that some of the inflammatory receptors were absent from the CSF-facing membrane and thus not activated by inclusion of the respective inflammatory marker.

## Conclusion

Here, we demonstrate that the CSF from iNPH patients contains elevated levels of a subset of inflammatory markers. Although the corresponding inflammatory receptors are, in general, expressed in the choroid plexus of rats and humans, their activation did not modulate the activity of the NKCC1 transport mechanism that is key in the CSF-secreting molecular machinery. Inflammation may possibly modulate other CSF-secreting transport mechanisms expressed in choroid plexus or other proposed CSF-secreting barriers, i.e. the endothelium or the ependymal cell layer. Unravelling of the molecular mechanisms underlying the pathological CSF accumulation seen in iNPH patients, and its relation to the inflammatory marker profile observed in this patient group, therefore awaits future research efforts.

## Supplementary Information

**Additional file 1: Table S1.** Inflammatory markers from Olink’s inflammation panel (Art. No. 95301). This table provides an overview of the inflammatory markers included in Olink’s inflammation panel and highlights the inflammatory markers excluded from the statistical analysis of the CSF samples

**Additional file 2: Table S2.** Statistical tests and results for the comparison of inflammatory markers levels iNPH patients and elderly control subjects.

**Additional file 3: Table S3.** Statistical tests and results for the comparison of inflammatory markers levels in iNPH responders and iNPH non-responders.

## Data Availability

The datasets used and/or analyzed during the current study are available from the corresponding author on reasonable request. The human choroid plexus data that supports the findings of this study are available in the GEO database, accession number GSE137619, https://www.ncbi.nlm.nih.gov/geo/query/acc.cgi?acc=GSE137619 [59].

## Abbreviations

ACSF: Artificial cerebrospinal fluid
CCL: C–C motif chemokine ligand
CCR: C–C motif chemokine receptor
CDCP1: CUB domain-containing protein 1
CSF: Cerebrospinal fluid
CXCL: C-X-C motif chemokine ligand
CXCR: C-X-C motif chemokine receptor
Flt3L: Fms-related tyrosine kinase 3 ligand
GEO: Gene Expression Omnibus
ICP: Intracranial pressure
IL: Interleukin
INPH: Idiopathic normal pressure hydrocephalus
LOD: Limit of detection
NKCC1: Na^+^/K^+^/2Cl^−^ cotransporter
NPX: Normalized protein expression
OPG: Osteoprotegerin
OSM: Oncostatin-M
PEA: Proximity extension assay
RNA-seq: RNA sequencing
TPM: Transcripts per million
VP: Ventriculo-peritoneal
4E-BP1: Eukaryotic translation initiation factor 4E-binding protein 1
