# Supplementary material for: Elevated CSF inflammatory markers in patients with idiopathic normal pressure hydrocephalus do not promote NKCC1 hyperactivity in rat choroid plexus
Source: Fluids Barriers CNS. 2021 Dec 4;18:54. doi: 10.1186/s12987-021-00289-6 (PMC8645122; doi:10.1186/s12987-021-00289-6)
Supplement: Supplementary file 1 — Additional file 1: Table S1. Inflammatory markers from Olink’s inflammation panel (Art. No. 95301). This table provides an overview of the inflammatory markers included in Olink’s inflammation panel and highlights the inflammatory markers excluded from the statistical analysis of the CSF samples [file 12987_2021_289_MOESM1_ESM.docx]

**Table S1**. Inflammatory markers from O-link’s inflammation panel (Art. No. 95301).

| **Inflammatory Markers** | |
| --- | --- |
| Adenosine Deaminase | ADA |
| **Artemin** | **ARTN** |
| **Axin-1** | **AXIN1** |
| Beta-nerve growth factor | Beta-NGF |
| **Caspase-8** | **CASP-8** |
| C-C motif chemokine 3 | CCL3 |
| C-C motif chemokine 4 | CCL4 |
| C-C motif chemokine 19 | CCL19 |
| **C-C motif chemokine 20** | **CCL20** |
| C-C motif chemokine 23 | CCL23 |
| C-C motif chemokine 25 | CCL25 |
| C-C motif chemokine 28 | CCL28 |
| CD40L receptor | CD40 |
| CUB domain-containing protein 1 | CDCP1 |
| C-X-C motif chemokine 1 | CXCL1 |
| C-X-C motif chemokine 5 | CXCL5 |
| C-X-C motif chemokine 6 | CXCL6 |
| C-X-C motif chemokine 9 | CXCL9 |
| C-X-C motif chemokine 10 | CXCL10 |
| C-X-C motif chemokine 11 | CXCL11 |
| Cystatin D | CST5 |
| Delta and Notch-like epidermal growth factor-related receptor | DNER |
| Eotaxin | CCL11 |
| Eukaryotic translation initiation factor 4E-binding protein 1 | 4E-BP1 |
| **Fibroblast growth factor 21** | **FGF-21** |
| **Fibroblast growth factor 23** | **FGF-23** |
| Fibroblast growth factor 5 | FGF-5 |
| Fibroblast growth factor 19 | FGF-19 |
| Fms-related tyrosine kinase 3 ligand | Flt3L |
| Fractalkine | CX3CL1 |
| **Glial cell line-derived neurotrophic factor** | **GDNF** |
| Hepatocyte growth factor | HGF |
| **Interferon gamma** | **IFN-gamma** |
| **Interleukin-1 alpha** | **IL-1 alpha** |
| **Interleukin-2** | **IL-2** |
| **Interleukin-2 receptor subunit beta** | **IL-2RB** |
| **Interleukin-4** | **IL-4** |
| **Interleukin-5** | **IL-5** |
| Interleukin-6 | IL-6 |
| Interleukin-7 | IL-7 |
| Interleukin-8 | IL-8 |
| **Interleukin-10** | **IL-10** |
| **Interleukin-10 receptor subunit alpha** | **IL-10RA** |
| Interleukin-10 receptor subunit beta | IL-10RB |
| Interleukin-12 subunit beta | IL-12B |
| **Interleukin-13** | **IL-13** |
| **Interleukin-15 receptor subunit alpha** | **IL-15RA** |
| **Interleukin-17A** | **IL-17A** |
| **Interleukin-17C** | **IL-17C** |
| Interleukin-18 | IL-18 |
| Interleukin-18 receptor 1 | IL-18R1 |
| **Interleukin-20** | **IL-20** |
| Interleukin-20 receptor subunit alpha | IL-20RA |
| **Interleukin-22 receptor subunit alpha-1** | **IL-22 RA1** |
| **Interleukin-24** | **IL-24** |
| **Interleukin-33** | **IL-33** |
| Latency-associated peptide transforming growth factor beta-1 | LAP TGF-beta-1 |
| Leukemia inhibitory factor | LIF |
| Leukemia inhibitory factor receptor | LIF-R |
| Macrophage colony-stimulating factor 1 | CSF-1 |
| Matrix metalloproteinase-1 | MMP-1 |
| Matrix metalloproteinase-10 | MMP-10 |
| Monocyte chemotactic protein 1 | MCP-1 |
| Monocyte chemotactic protein 2 | MCP-2 |
| **Monocyte chemotactic protein 3** | **MCP-3** |
| Monocyte chemotactic protein 4 | MCP-4 |
| Natural killer cell receptor 2B4 | CD244 |
| **Neurotrophin-3** | **NT-3** |
| **Neurturin** | **NRTN** |
| Oncostatin-M | OSM |
| Osteoprotegerin | OPG |
| Programmed cell death 1 ligand 1 | PD-L1 |
| **Protein S100-A12** | **EN-RAGE** |
| **Signaling lymphocytic activation molecule** | **SLAMF1** |
| SIR2-like protein 2 | SIRT2 |
| STAM-binding protein | STAMBP |
| Stem cell factor | SCF |
| **Sulfotransferase 1A1** | **ST1A1** |
| **T cell surface glycoprotein CD6 isoform** | **CD6** |
| T-cell surface glycoprotein CD5 | CD5 |
| T-cell surface glycoprotein CD8 alpha chain | CD8A |
| **Thymic stromal lymphopoietin** | **TSLP** |
| TNF-beta | TNFB |
| **TNF-related activation-induced cytokine** | **TRANCE** |
| TNF-related apoptosis-inducing ligand | TRAIL |
| Transforming growth factor alpha | TGF-alpha |
| Tumor necrosis factor ligand superfamily, member 12 | TWEAK |
| **Tumor necrosis factor** | **TNF** |
| Tumor necrosis factor ligand superfamily member 14 | TNFSF14 |
| Tumor necrosis factor receptor superfamily member 9 | TNFRSF9 |
| Urokinase-type plasminogen activator | uPA |
| Vascular endothelial growth factor A | VEGF-A |

The inflammatory markers highlighted in **bold** were excluded from the statistical analysis as their cerebrospinal fluid levels were below the limit of detection in more than 35 % of the samples.
