# Supplementary material for: Elevated CSF inflammatory markers in patients with idiopathic normal pressure hydrocephalus do not promote NKCC1 hyperactivity in rat choroid plexus
Source: Fluids Barriers CNS. 2021 Dec 4;18:54. doi: 10.1186/s12987-021-00289-6 (PMC8645122; doi:10.1186/s12987-021-00289-6)
Supplement: Supplementary file 2 — Additional file 2: Table S2. Statistical tests and results for the comparison of inflammatory markers levels iNPH patients and elderly control subjects. [file 12987_2021_289_MOESM2_ESM.docx]

**Table S2.** Statistical tests and results for the comparison of inflammatory markers levels iNPH patients and elderly control subjects.

| **Inflammatory marker** | **Statistical Test** | **P-value** |
| --- | --- | --- |
| CCL28 | Mann-Whitney | 0.0007 |
| CCL23 | Mann-Whitney | 0.0051 |
| CCL3 | Mann-Whitney | 0.0056 |
| OPG | T-test | 0.0207 |
| CXCL1 | Mann-Whitney | 0.0211 |
| CDCP1 | T-test | 0.0225 |
| IL18 | Mann-Whitney | 0.0227 |
| IL8 | Mann-Whitney | 0.0263 |
| OSM | Mann-Whitney | 0.0283 |
| 4E-BP1 | Mann-Whitney | 0.0283 |
| CXCL6 | Mann-Whitney | 0.0375 |
| Flt3L | Mann-Whitney | 0.0402 |
| CCL25 | Mann-Whitney | 0.0718 |
| TNFRSF9 | Mann-Whitney | 0.0763 |
| CXCL5 | Mann-Whitney | 0.0859 |
| FGF-5 | T-test | 0.0876 |
| CXCL9 | Mann-Whitney | 0.0911 |
| CCL11 | Mann-Whitney | 0.1274 |
| STAMBP | Mann-Whitney | 0.1274 |
| IL-18R1 | T-test | 0.1936 |
| PD-L1 | Mann-Whitney | 0.2012 |
| DNER | Mann-Whitney | 0.2211 |
| TWEAK | T-test | 0.2615 |
| CD244 | Mann-Whitney | 0.2766 |
| TNFB | Mann-Whitney | 0.2766 |
| HGF | T-test | 0.2792 |
| LIF | Mann-Whitney | 0.2888 |
| TNFSF14 | Mann-Whitney | 0.3141 |
| MCP-1 | T-test | 0.3188 |
| Beta-NGF | T-test | 0.3671 |
| IL7 | Mann-Whitney | 0.3834 |
| CD5 | Mann-Whitney | 0.3983 |
| CCL4 | Mann-Whitney | 0.4135 |
| TRAIL | Mann-Whitney | 0.4291 |
| CCL19 | T-test | 0.4354 |
| TGF-alpha | T-test | 0.4703 |
| IL6 | Mann-Whitney | 0.4777 |
| FGF-19 | Mann-Whitney | 0.4945 |
| ADA | T-test | 0.5075 |
| LAP TGF-beta-1 | T-test | 0.5140 |
| IL-10RB | Mann-Whitney | 0.5291 |
| VEGFA | T-test | 0.5337 |
| MMP-1 | Mann-Whitney | 0.5648 |
| IL-12B | Mann-Whitney | 0.5648 |
| uPA | Mann-Whitney | 0.5831 |
| MMP-10 | Mann-Whitney | 0.6017 |
| CXCL11 | Mann-Whitney | 0.6395 |
| CX3CL1 | Mann-Whitney | 0.6783 |
| SCF | T-test | 0.7444 |
| IL-20RA | T-test | 0.7875 |
| CXCL10 | Mann-Whitney | 0.8201 |
| LIF-R | T-test | 0.8336 |
| CST5 | Mann-Whitney | 0.8620 |
| MCP-4 | Mann-Whitney | 0.8620 |
| CSF-1 | T-test | 0.8714 |
| SIRT2 | T-test | 0.9438 |
| CD40 | Mann-Whitney | 0.9680 |
| MCP-2 | Mann-Whitney | 0.9893 |
| CD8A | Mann-Whitney | 1 |

See Methods for details on choice of test.
