# Supplementary material for: Elevated CSF inflammatory markers in patients with idiopathic normal pressure hydrocephalus do not promote NKCC1 hyperactivity in rat choroid plexus
Source: Fluids Barriers CNS. 2021 Dec 4;18:54. doi: 10.1186/s12987-021-00289-6 (PMC8645122; doi:10.1186/s12987-021-00289-6)
Supplement: Supplementary file 3 — Additional file 3: Table S3. Statistical tests and results for the comparison of inflammatory markers levels in iNPH responders and iNPH non-responders. [file 12987_2021_289_MOESM3_ESM.docx]

**Table S3**. Statistical tests and results for the comparison of inflammatory markers levels in iNPH responders and iNPH non-responders.

| **Inflammatory marker** | **Statistical Test** | **P-value** |
| --- | --- | --- |
| STAMBP | T-test | 0.0937 |
| MMP-10 | Mann-Whitney | 0.1431 |
| CXCL6 | T-test | 0.1604 |
| CXCL10 | T-test | 0.1770 |
| CXCL11 | T-test | 0.1885 |
| MCP-4 | Mann-Whitney | 0.1903 |
| CXCL5 | Mann-Whitney | 0.1903 |
| CST5 | T-test | 0.1930 |
| FGF-19 | Mann-Whitney | 0.2176 |
| LAP TGF-beta-1 | T-test | 0.2500 |
| CX3CL1 | T-test | 0.2685 |
| Beta-NGF | T-test | 0.2831 |
| IL-12B | T-test | 0.3009 |
| MCP-1 | Mann-Whitney | 0.3150 |
| CD40 | T-test | 0.3153 |
| CSF-1 | T-test | 0.3217 |
| MCP-2 | T-test | 0.3230 |
| IL6 | T-test | 0.3238 |
| FGF-5 | T-test | 0.3461 |
| CCL28 | T-test | 0.3516 |
| CD8A | T-test | 0.3729 |
| PD-L1 | Mann-Whitney | 0.3930 |
| TRAIL | T-test | 0.4207 |
| SCF | T-test | 0.4545 |
| TGF-alpha | T-test | 0.4645 |
| VEGFA | T-test | 0.4758 |
| 4E-BP1 | T-test | 0.4771 |
| DNER | T-test | 0.4787 |
| CCL19 | T-test | 0.4812 |
| CCL23 | T-test | 0.4963 |
| CCL4 | T-test | 0.5116 |
| IL-10RB | T-test | 0.6129 |
| OPG | T-test | 0.6175 |
| uPA | Mann-Whitney | 0.6305 |
| TNFB | Mann-Whitney | 0.6305 |
| TWEAK | T-test | 0.6369 |
| CCL25 | T-test | 0.6595 |
| CDCP1 | T-test | 0.6718 |
| ADA | T-test | 0.6733 |
| HGF | T-test | 0.6875 |
| SIRT2 | T-test | 0.7013 |
| TNFRSF9 | T-test | 0.7206 |
| CXCL1 | Mann-Whitney | 0.7394 |
| TNFSF14 | Mann-Whitney | 0.7394 |
| CXCL9 | T-test | 0.7608 |
| IL8 | T-test | 0.7673 |
| IL18 | T-test | 0.7861 |
| CD244 | T-test | 0.8018 |
| IL-18R1 | T-test | 0.8370 |
| CCL3 | T-test | 0.8424 |
| MMP-1 | Mann-Whitney | 0.8534 |
| CD5 | T-test | 0.8685 |
| LIF-R | Mann-Whitney | 0.9118 |
| Flt3L | T-test | 0.9487 |
| IL-20RA | T-test | 0.9649 |
| IL7 | T-test | 0.9666 |
| LIF | T-test | 0.9700 |
| CCL11 | T-test | 0.9861 |
| OSM | T-test | 0.9926 |

See Methods for details on choice of test.
